# Risk factors and nomogram-based prediction of the risk of limb weakness in herpes zoster

**DOI:** 10.3389/fnins.2023.1109927

**Published:** 2023-03-13

**Authors:** Shao-jun Li, Dan Feng

**Affiliations:** Department of Pain Management, Wuhan No.1 Hospital, Wuhan, Hubei, China

**Keywords:** risk factors, nomogram, limb weakness, herpes zoster, treatment

## Abstract

**Background:**

Limb weakness is a less common complication of herpes zoster (HZ). There has been comparatively little study of limb weakness. The aim of this study is to develop a risk nomogram for limb weakness in HZ patients.

**Methods:**

Limb weakness was diagnosed using the Medical Research Council (MRC) muscle power scale. The entire cohort was assigned to a training set (from January 1, 2018 to December 30, 2019, *n* = 169) and a validation set (from October 1, 2020 to December 30, 2021, *n* = 145). The least absolute shrinkage and selection operator (LASSO) regression analysis method and multivariable logistic regression analysis were used to identify the risk factors of limb weakness. A nomogram was established based on the training set. The discriminative ability and calibration of the nomogram to predict limb weakness were tested using the receiver operating characteristic (ROC) curve, calibration plots, and decision curve analysis (DCA). A validation set was used to further assess the model by external validation.

**Results:**

Three hundred and fourteen patients with HZ of the extremities were included in the study. Three significant risk factors: age (OR = 1.058, 95% CI: 1.021–1.100, *P* = 0.003), VAS (OR = 2.013, 95% CI: 1.101–3.790, *P* = 0.024), involving C6 or C7 nerve roots (OR = 3.218, 95% CI: 1.180–9.450, *P* = 0.027) were selected by the LASSO regression analysis and the multivariable logistic regression analysis. The nomogram to predict limb weakness was constructed based on the three predictors. The area under the ROC was 0.751 (95% CI: 0.673–0.829) in the training set and 0.705 (95% CI: 0.619–0.791) in the validation set. The DCA indicated that using the nomogram to predict the risk of limb weakness would be more accurate when the risk threshold probability was 10–68% in the training set and 15–57% in the validation set.

**Conclusion:**

Age, VAS, and involving C6 or C7 nerve roots are potential risk factors for limb weakness in patients with HZ. Based on these three indicators, our model predicted the probability of limb weakness in patients with HZ with good accuracy.

## Introduction

Herpes zoster (HZ) is a painful skin disease caused by the reactivation of the latent varicella-zoster virus (VZV). It appears as a vesicular rash with a dermatomal distribution (Pan et al., 2022). Postherpetic neuralgia is a common complication of HZ. Persistent pain can have a negative impact on daily life and increase the financial burden. Limb weakness is a less common complication of HZ. The VZA damages nerve in the upper or lower limbs, causing myotomal motor weakness or paralysis. HZ-related limb weakness is distinguished by focal, asymmetrical dyskinesia in the myotome corresponding to the rash’s dermatome (Ruppert et al., 2010). This disease is much more prevalent in the elderly, diabetes mellitus, and immunodeficient patients. Weakness does not occur in all patients with limb HZ. In the early stages of HZ, as the pain is controlled, limb weakness can be self-limited. With time, limb weakness may become difficult to treat. Even if the pain is relieved, the strength of the limbs cannot be restored to normal levels. Long-term persistent limb weakness can lead to physical disability, reduce the quality of life, and raise the economic burden.

Early diagnosis of limb weakness in HZ is difficult. The majority of HZ patients have no specific clinical manifestations. Previous research has found that magnetic resonance imaging (MRI) and electrophysiologic diagnosis have a high sensitivity for detecting motor impairments (Santiago-Pérez et al., 2012). Reda et al. (2012) reviewed eight HZ patients who had segmental paresis. Four patients had MRI scans and all affected nerves in the MRI were enlarged. Affected nerves had abnormalities that extended longitudinally along the length of the nerve. All the patients had electrophysiologic changes of mononeuropathies. Jones et al. (2014) reviewed 49 HZ patients with zoster-associated limb paresis. Twenty-six patients underwent MRI examination. Nerve imaging abnormalities were found in 19 patients. An electrodiagnostic examination revealed that 47 of the 49 patients had fibrillation potentials. Electrophysiologic changes were present in all of the patients. However, although imaging and electrophysiologic examinations are helpful in diagnosing limb weakness in HZ, they are not specific. A retrospective analysis of 10 patients with zoster-associated limb paresis found that imaging abnormalities were found in 7 patients. 50% of patients presented with secondary denervation changes in shoulder girdle muscles and nerve T2 signal hyperintensity. 20% of patients had nerve enlargement (Zubair et al., 2017). Mondelli et al. (2002) have reported a prospective case series with 158 HZ patients. 36% of patients had electromyographic signs of abnormal spontaneous activity. Motor fiber damage was evident clinically in 19% (segmental zoster paresis) and was subclinical and detectable only by electromyography in 17%. MRI and electrophysiologic examination may be normal in the early stages of the disease. Once limb weakness appears, treatment can be extremely difficult. Furthermore, previous studies have had relatively small sample sizes and have focused on describing clinical characteristics, electrophysiologic, and imaging features. In this study, we retrospectively analyzed 314 limb HZ patients and identified risk factors for limb weakness in HZ patients. In particular, we sought to create a prediction model that can quickly identify patients at risk of limb weakness in the HZ.

## Materials and methods

### Patient selection

This retrospective study was approved by the Medical Ethics Committee of the Wuhan No 1 Hospital. All patients provided written informed consent. The records of 330 patients with limbs HZ were reviewed for the study. The entire cohort was assigned to a training set (from January 1, 2018 to December 30, 2019, *n* = 169) and a validation set (from October 1, 2020 to December 30, 2021).

### Inclusion and exclusion

The inclusion criteria were as follows: (1) limbs HZ pain; (2) inpatient treatment (i.e., when VAS ≥ 4 and disease course ≥ 1 week); (3) age ≥ 18 years or ≤85 years; (4) having a good understanding of Chinese.

**FIGURE 1:**
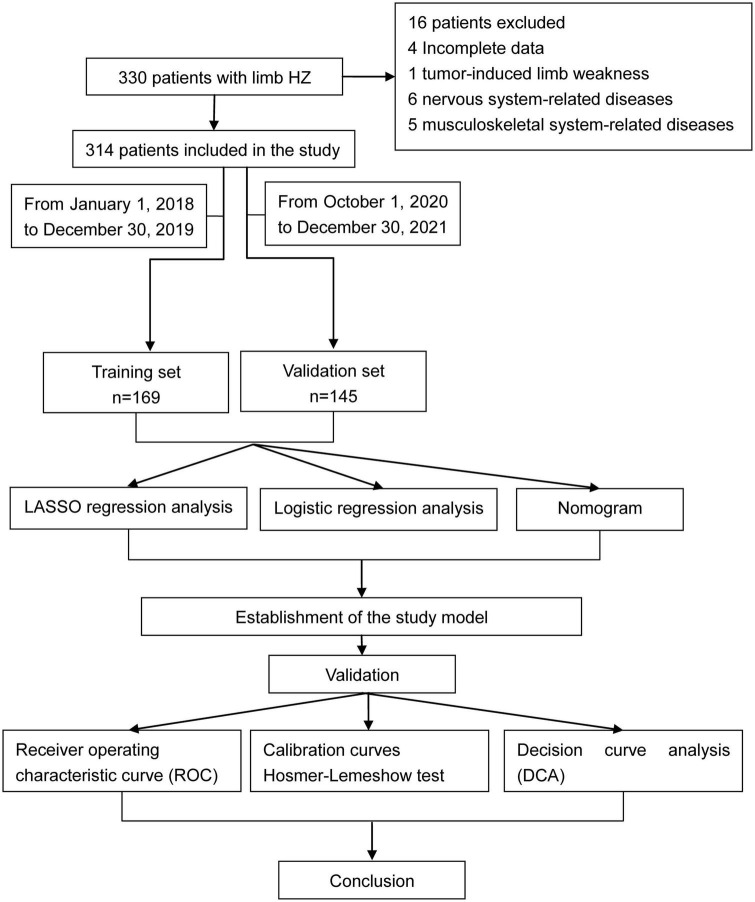
Flowchart of the study.

The exclusion criteria were as follows: (1) HZ located in the head, neck, trunk, and perineum area; (2) tumor-induced limb weakness; (3) nervous system-related diseases; (4) musculoskeletal system-related diseases; (5) incomplete data; (6) refusing to participate in the study (Figure 1).

### Data collection and follow-up

This is a retrospective study conducted with the aid of an electronic medical record system. Data were gathered using an electronic medical record system, the telephone, letters, and e-mail. We created a dedicated database in which we recorded demographic data (such as age, and gender), individual disease history, VAS, rash site, course of HZ, history of the HZ episode, location of skin lesions, disease stage and severity, immune status, and clinical treatments. All patients had electrophysiologic evidence of nerve injury in the study. Limb weakness was diagnosed using the Medical Research Council (MRC) muscle power scale, which ranks muscle power from 0 to 5 (0: no muscle contraction; (1): muscle contraction but no joint activity; (2): full range movement of joint under no gravity; (3): full range movement of joint under anti-gravity state; (4): joint can resist partial resistance activity, but worse than normal; (5): normal muscle strength, that is, joint can achieve full range movement under maximum resistance). Muscle weakness is defined as muscle strength below grade 5 and lower than that of the contralateral limb (normal side). Muscle strength equal to or less than grade 5, but the same as the contralateral limb, was not recognized as muscle weakness (Medical Research Council, 1981). The intensity of pain was measured using a VAS scale ranging from 0 to 10 (0 represents no pain, and 10 represent the most severe pain). Questionnaires for muscle weakness and pain scores were completed by a physician at admission and 3 months after discharge.

### Statistical analyses

R software (version 4.2.1; R Foundation for Statistical Computing, Vienna, Austria) was used for statistical analyses. Demographic and clinical data for patients with limbs HZ were characterized using mean ± SD and count (percentage), respectively, to represent continuous and categorical factors. The least absolute shrinkage and selection operator (LASSO) regression analysis was performed to analyze the data in the training set in order to identify the best predictors among the candidate risk variables. The “glmnet” package of R was used to run LASSO regression analysis. The optimal hyper-parameter lambda of the LASSO model was determined through a K-fold cross-validation procedure repeated 10 times by identifying the largest lambda value that obtained an error within one standard deviation from the minimum binomial deviance (“lambda.1se”). The number K of folds used for the cross-validation was 10. “Lambda.1se” provides a model with good performance and a minimal set of independent variables. The multivariable logistic regression analysis was used to determine the risk factors of limb weakness in HZ patients. The predicting model was formulated based on the results of the multivariable logistic regression analysis.

A nomogram was created based on the results of multivariate analysis using the “rms” R software package. The nomogram maps the predicted probabilities as points on a scale from 0 to 100. The total score accumulated by the various covariates corresponds to the predicted probability of the patient. The pROC package was used to plot the receiver operating characteristic (ROC) curve and to compute the area under the curve (AUC) of the predictive model. Hosmer–Lemeshow goodness-of-fit test and a calibration curve were used to calibrate the model. A 45-degree diagonal line indicates the perfect calibration line. The calibration line, and deviations above or below this line reflect under-prediction or over-prediction. The decision curve analysis (DCA) was performed to quantify the clinical practicability of nomograms based on the net benefit under different threshold probabilities. By using the data of the validation set, ROC, calibration curve, DCA were used to estimate the accuracy of the risk prediction model.

## Results

### Baseline characteristics

The entire cohort was assigned to a training set (from January 1, 2018 to December 30, 2019, *n* = 169) and a validation set (from October 1, 2020 to December 30, 2021, *n* = 145).

Ninety-six (30.6%) HZ patients presented with limb weakness (53 out of 169 in the training set; 43 out of 145 in the validation set). The proportion of male and female patients with HZ was similar in the two sets. Forty-four (14.0%) HZ patients had more than twice hospitalizations. The vast majority of the patients (69.1%) had a course of disease longer than 14 days. Table 1 shows the baseline characteristics of participants in the training set and validation set.

**TABLE 1:** Baseline characteristics of participants in the training set and validation set.

Items	Whole cohort (*n* = =314)	Training set (*n* = =169)	Validation set (*n* = =145)
Age, year, Mean ± SD	66.8 ± 10.6	66.5 ± 10.6	67.2 ± 10.7
**Gender, n (%)**			
Men	149(47.5)	77(45.6)	72(49.7)
Women	165(52.5)	92(54.4)	73(50.3)
**Times of hospitalization**			
Once	270(86.0)	147(87.0)	123(84.8)
More than twice	44(14.0)	22(13.0)	22(15.2)
Course, day, Mean ± SD	45.1 ± 110.6	37.3 ± 102.4	54.2 ± 119.2
**Side, n (%)**			
Left	159(50.6)	85(50.3)	74(51.0)
Right	155(49.4)	84(49.7)	71(49.0)
VAS, Mean ± SD	6.17 ± 0.67	6.08 ± 0.61	6.26 ± 0.73
**Diabetes, n (%)**			
Yes	64(20.4)	35(20.7)	29(20.0)
No	250(79.6)	134(79.3)	116(80.0)
**Comorbiditiy^&^, n (%)**			
<3	273(86.9)	146(86.4)	127(87.6)
≥3	41(13.1)	23(13.6)	18(12.4)
**Malignant tumor, n (%)**			
Yes	16(5.1)	12(7.1)	4(2.8)
No	298(94.9)	157(92.9)	141(97.2)
**Rash site, n (%)**			
Upper limbs	163(51.9)	87(51.5)	76(52.4)
Lower limbs	151(48.1)	82(48.5)	69(47.6)
C6 or C7 level*, n (%)			
Yes	97(30.9)	56(33.1)	41(28.3)
No	217(69.1)	113(66.9)	104(71.7)

^&^Conditions other than diabetes, such as hypertension, coronary heart disease, pulmonary disease, etc.

*Involving C6 or C7 nerve roots.

### Variables selection

**FIGURE 2:**
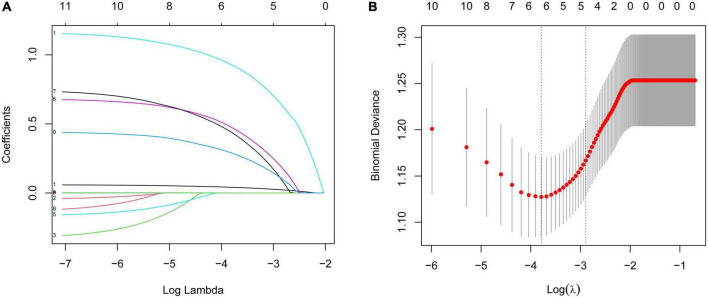
**(A)** Variable selection by LASSO regression model. A coefficient profile plot was produced against the log(lambda) sequence. Each curve corresponds to a variable. It shows the path of its coefficient against the L1-norm of the whole coefficient vector at various λ values. **(B)** Partial likelihood deviance for the LASSO coefficient profiles. The red dotted line stands for the cross-validation curve, error bars represent the upper and lower standard deviation curves along the λ sequence. Five variables with non-zero coefficients were found by optimal λ (λ = 0.05527638).

All the variables (including age, gender, and times of hospitalization, course, side, VAS, diabetes, comorbidity, malignant tumor, rash site, and HZ involving C6 or C7 nerve roots) were evaluated by the LASSO regression analysis. The variables selected by optimal lambda (λ) are the simplest model with the highest performance. The optimal penalty parameter λ was 0.05527638. Five significant risk factors (including age, VAS, involving C6 or C7 nerve roots, diabetes, and course) were selected by the LASSO regression analysis (Figures 2A, B). Then the selected factors were analyzed by multivariable logistic regression. The results showed that age (OR = 1.058, 95% CI: 1.021–1.100, *P* = 0.003), VAS (OR = 2.013, 95% CI: 1.101–3.790, *P* = 0.024), involving C6 or C7 nerve roots (OR = 3.218, 95% CI: 1.180–9.450, *P* = 0.027) were independent predictors for limbs weakness in patients with HZ (Table 2).

**TABLE 2:** Multivariable logistic regression analysis of limb weakness in HZ patients.

Items	OR	95% CI	*P*-value
Age, years VAS C6 or C7 level* (yes or no)	1.058 2.013 3.218	1.021–1.100 1.101–3.790 1.180–9.450	0.003 0.024 0.027

*Involving C6 or C7 nerve roots.

### Predictive model construction

**FIGURE 3:**
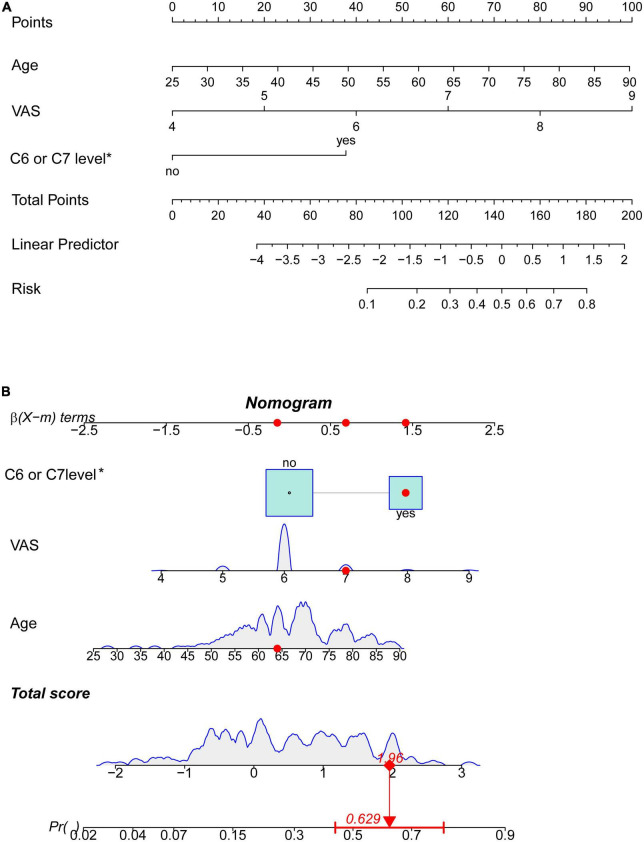
Nomogram prediction model (*involving C6 or C7 nerve roots). **(A)** Nomogram based on three risk factors to predict limb weakness probabilities of HZ patients. **(B)** The dynamic nomogram for an example. An HZ patient with less than 64 years, a VAS of 7, and involving C6 or C7 nerve roots has an estimated probability of limb weakness of 62.9%.

Figure 3A showed a nomogram to predict limb weakness of HZ patients. The nomogram to predict limb weakness was constructed based on the three predictors: age, VAS, and HZ involving C6 or C7 nerve roots. Total points were calculated by adding the number of points allocated to each factor in the nomogram. A high total score indicated a high probability of limb weakness. For example, an HZ patient with 64 years, a VAS of 7, and HZ involving C6 or C7 nerve roots has an estimated probability of limb weakness of 62.9% (Figure 3B).

### Predictive model validation

**FIGURE 4:**
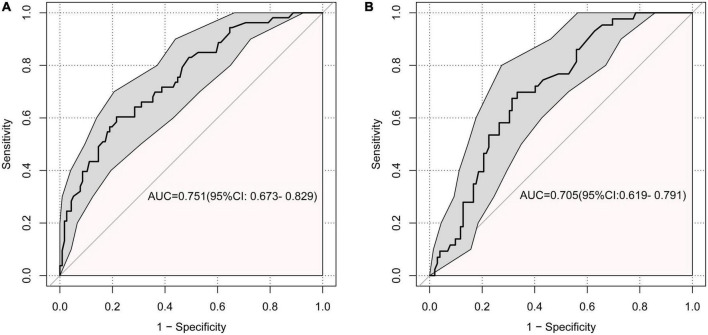
Receiver operating characteristic curve (ROC) for the discrimination of the model. **(A)** The training set, 0.751 (95% CI: 0.673–0.829). **(B)** The validation set, 0.705 (95% CI: 0.619–0.791).

For the training set, the area under the ROC was 0.751 (95% CI: 0.673–0.829), indicating good discrimination. While the area under the ROC of the validation set was 0.705 (95% CI: 0.619–0.791) indicating acceptable discriminative power (Figures 4A, B).

**FIGURE 5:**
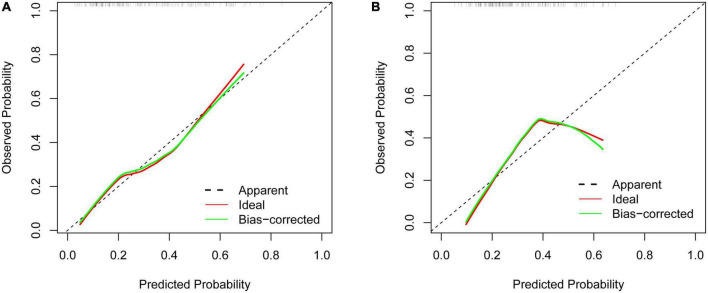
Calibration curves of the predictive limb weakness risk nomogram. **(A)** The training set. **(B)** The validation set.

The calibration curve showed that there was good agreement between the predicted and observed probabilities in both the training set and the validation set (Figures 5A, B). Hosmer–Lemeshow test was also used to calibrate the model. The results showed good agreement in the training sets (*P* = 0.783).

**FIGURE 6:**
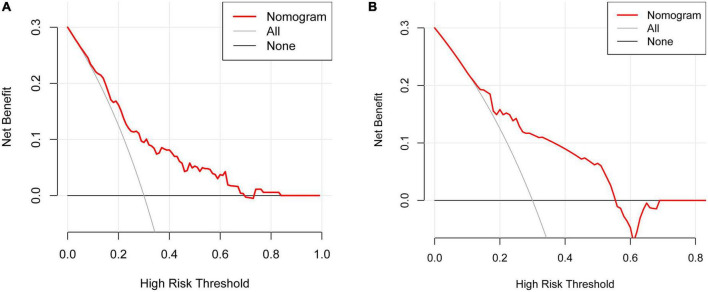
Decision curve analysis (DCA) for the limb weakness risk nomogram. The thick solid line indicated the assumption that no limb weakness existed in any of the patients. The thin solid line denoted the idea that all patients suffered from limb weakness. The risk nomogram was represented by the red line. **(A)** The training set. **(B)** The validation set.

The DCA indicated that using the nomogram to predict the risk of limb weakness would be more accurate when the risk threshold probability was 10–68% in the training set and 15–57% in the validation set (Figures 6A, B).

## Discussion

Limb weakness is a relatively rare neurological complication of HZ. The rate of limb weakness ranged from 0.3 to 5% (Gupta et al., 1969; Thomas and Howard, 1972). The true incidence of this neurological complication was underestimated due to the atypical clinical symptoms and difficulty in clinical diagnosing. Anterior spinal roots, anterior horn cells, the brachial or lumbar plexus, and peripheral nerves may be the injury sites. MRI and electrophysiologic examinations help to correctly diagnose and assess the extent of the lesion.

The mechanisms of limb weakness in patients with HZ are not completely understood. In the past, the sites of motor injury were believed to occur at the level of the root or anterior horn cell. Studies have found that viral spread from the dorsal root ganglion to the adjacent nerve tissue, including the anterior horn cells and motor roots (Ismail et al., 2009; Ayoub et al., 2010). More recent studies have reported that axonal pathology may be the responsible mechanism for limb weakness. Reda et al. (2012) reported 8 patients with zoster-associated limb paresis. Electrophysiologic examination revealed that all the patients had fibrillation potentials in affected muscles. This suggested that motor injury was associated with axonal loss, resulting in nerve conduction block. While Worrell and Cockerell (1997) have reported that VZV infected the peripheral and central nervous systems cells, leading to peri- and intraneural inflammation as well as mononuclear or leukocytoclastic vasculitis. In addition, the pathological injury at the anterior root, anterior horn cells, brachial plexus, lumbar plexus, and peripheral nerves was related to zoster-associated limb paresis (Dobrev et al., 2008; Tashiro et al., 2010). These mechanisms are mainly speculative, with conflicting opinions and a lack of histological basis. Therefore, further studies are needed.

The present study established a nomogram for the prediction of the risk of limb weakness in patients with HZ. Age, VAS, and involving C6 or C7 nerve roots are independent risk factors for limb weakness in patients with HZ.

Age is risk factors for limb weakness in patients with HZ. Age is significantly associated with the incidence of limb weakness. Studies have found that the mean age of segmental zoster paresis was 69 years old. The highest incidence was 60–70 years old (Liu et al., 2018; Meng et al., 2021). The elderly are more vulnerable to VZV and higher motor complications than children and young adults. A 6-year population-based analysis of HZ has found that postherpetic neuralgia risk was increased with age. The cumulative risk of developing postherpetic neuralgia between ages 50–90 years was 6.9 (95% CI: 6.7–7.1) (Muñoz-Quiles et al., 2018). Reda et al. (2012) believed that segmental zoster paresis was associated with a high rate of postherpetic neuralgia. Therefore, physicians should be alert to the need for early intervention of HZ in the extremities in the elderly.

Pain intensity is also related to the occurrence of limb weakness. Pain is the main clinical manifestation of HZ and postherpetic neuralgia. One study reported the relationship between pain and limb weakness. They concluded that the numerical rating scale (NRS) score was not related to motor dysfunction of limb HZ (Tang et al., 2022). Our study reported, on the contrary, the opposite. A higher VAS at onset is a risk factor for developing zoster paresis of limbs. Numerous studies have demonstrated that serious pain has been found to correlate with poor quality of life (Bowsher, 1997; Pickering and Leplege, 2011; Van Wijck and Aerssens, 2017). Pain occurring in the extremities may prevent the patient from moving the upper or lower extremities. Long-term inactivity of the limbs can aggravate limb weakness, even leading to muscle atrophy. It should be noted that severe pain can cause temporary paralysis in patients who are reluctant to move their limbs. Some patients had a full return of limb function after pain relief. Therefore, reducing pain helps in limb effective rehabilitation.

The upper limb HZ, particularly involving C6 or C7 segments, was strongly connected with the incidence of limb weakness. Segmental zoster paresis occurrence on the upper extremities seemed to be higher than lower extremities in several case reports in the literature (Yoleri et al., 2005; Rastegar et al., 2015; Teo et al., 2016; Chen et al., 2020; Patel et al., 2022). Liu et al. (2018) found that limb weakness occurred in 6 out of 8 patients with upper limbs HZ. A previous study has revealed that under half (22/49) of the paresis affected the upper limb (Jones et al., 2014). There have been reports of both upper and lower extremities being the same incidence (Thomas and Howard, 1972; Molloy and Goodwill, 1979). However, the results of these studies were not statistically significant. Tang et al. (2022) found that motor dysfunction was more likely to occur in HZ patients with upper limb involvement (95% CI: 1.829–8.387, *P* = 0.001). Our study supports this conclusion. The HZ involving C6 or C7 segments is more likely to develop motor weakness. A study retrospectively analyzing 87 cases of upper extremity weakness from 1961- to 2007 showed that weakness was most frequent in the proximal muscles or C5–C7 segments (Kawajiri et al., 2007). Therefore, physicians should pay high attention to the upper limbs HZ, especially involving C6 or C7 segments patients.

The DCA is used to determine the clinical practicability of nomograms based on the net benefit under different threshold probability in patients with HZ on the extremities. According to the DCA in the training set, clinicians can use the following methods when they make decisions using predictive models: (1) A specific threshold probability was set for all patients (e.g., 10%). If the model predicts a value greater than this probability, treatment of limb weakness is performed, and if less, no treatment is performed; (2) All patients with herpes zoster of the extremities were divided into low- and medium-risk and high-risk groups; the high-risk group was treated for limb weakness, the low-risk group was not, and the medium-risk group was specifically analyzed according to age, systemic condition, and patient wishes. It should be noted that the thresholds used for identifying the three proposed risk groups have to be decided by the clinicians that want to use the nomogram; (3) An individualized threshold is determined based on communication with the patient, and this threshold is then compared with the model predicted value. If the predicted value is greater than the threshold, treatment of limb weakness is performed, and vice versa. In short, this model can provide an idea and a therapeutic direction for clinicians to treat limb weakness early. Early detection and early intervention can alleviate the decline in quality of life caused by limb weakness. However, application of the model in clinical practice should pay attention to individualized treatment because of the proposed predictive model obtaining modest accuracy.

Nomogram is widely used in both oncologic and non-oncologic diseases (Huang et al., 2022; Zan et al., 2022; Zhang et al., 2022). Nomograms can create a simple graphical representation of a statistical predictive model that generates a numerical probability of a clinical event (Iasonos et al., 2008). The ability of nomograms to combine multiple important prognostic factors to quantify individual risk enables it more acceptable to physicians and patients. We construct a model to predict the risk of upper limb weakness in HZ patients. The LASSO regression analysis was used to resolve the problem of multicollinearity in the variables. According to the time of enrollment in the study, we divided patients into two groups for external verification. The results of ROC, calibration and DCA curves for the training set and validation set verified the accuracy and stability of the model.

Our study has several limitations. First, the number of limb weakness factors and the sample size are relatively small. We mainly focused on clinical characteristics and lacked laboratory data. Second, we did not assess the MRI and electrophysiological abnormalities in the HZ patients. Third, the Medical Research Council (MRC) muscle power system and VAS are subjective measurements for the assessment of muscle power and pain intensity.

## Conclusion

Age, VAS, and involving C6 or C7 nerve roots are potential risk factors for limb weakness in patients with HZ. Based on these three indicators, our model predicted the probability of limb weakness in patients with HZ with good accuracy.

## Data availability statement

The raw data supporting the conclusions of this article will be made available by the authors, without undue reservation.

## Ethics statement

The studies involving human participants were reviewed and approved by the Wuhan No.1 Hospital. The patients/participants provided their written informed consent to participate in this study.

## Author contributions

DF: conceptualization, methodology, supervision, software, and validation. S-JL: data curation, writing—original draft preparation, visualization, investigation, and writing—review and editing. Both authors contributed to the article and approved the submitted version.
